# Obstructive Sleep Apnea and Dementia-Common Gene Associations through Network-Based Identification of Common Driver Genes

**DOI:** 10.3390/genes12040542

**Published:** 2021-04-09

**Authors:** Hyun-Hwan Jeong, Arvind Chandrakantan, Adam C. Adler

**Affiliations:** 1 Department of Molecular and Human Genetics, Baylor College of Medicine, Houston, TX 77030, USA; 2 Department of Anesthesiology & Pediatrics, Texas Children’s Hospital, Houston, TX 77030, USA; adam.adler@bcm.edu; 3 Department of Anesthesiology, Baylor College of Medicine, Houston, TX 77030, USA

**Keywords:** Obstructive Sleep Apnea, driver genes, Alzheimer’s disease

## Abstract

Background: Obstructive Sleep Apnea (OSA) occurs in 7% of the adult population. The relationship between neurodegenerative diseases such as dementia and sleep disorders have long attracted clinical attention; however, no comprehensive data exists elucidating common gene expression between the two diseases. The objective of this study was to (1) demonstrate the practicability and feasibility of utilizing a systems biology approach called network-based identification of common driver genes (NICD) to identify common genomic features between two associated diseases and (2) utilize this approach to identify genes associated with both OSA and dementia. Methods: This study utilized 2 public databases (PCNet, DisGeNET) and a permutation assay in order to identify common genes between two co-morbid but mutually exclusive diseases. These genes were then linked to their mechanistic pathways through Enrichr, producing a list of genes that were common between the two different diseases. Results: 42 common genes were identified between OSA and dementia which were primarily linked to the G-coupled protein receptor (GPCR) and olfactory pathways. No single nucleotide polymorphisms (SNPs) were identified. Conclusions: This study demonstrates the viability of using publicly available databases and permutation assays along with canonical pathway linkage to identify common gene drivers as potential mechanistic targets for comorbid diseases.

## 1. Introduction

**Figure 1:**
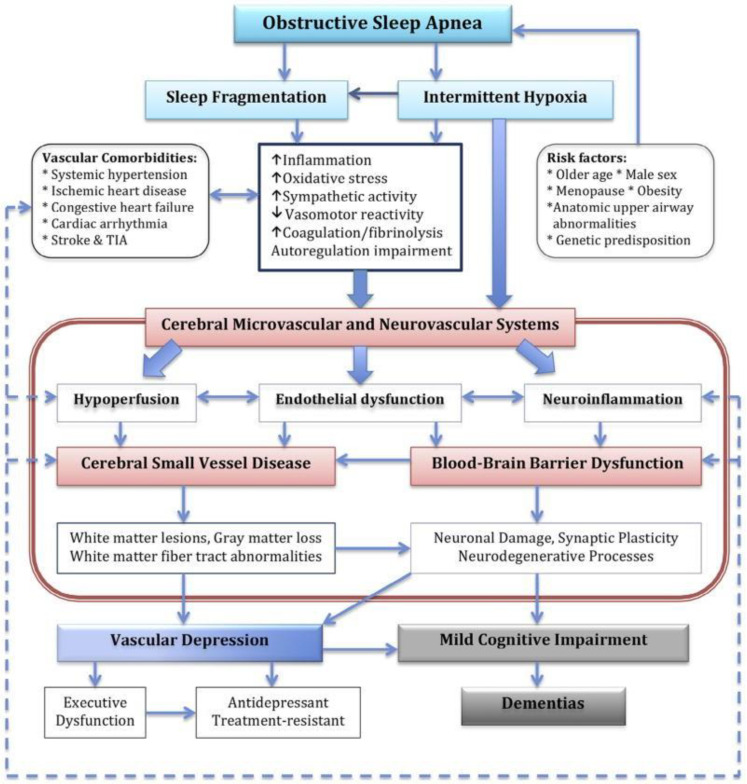
Describes the variety of pathophysiological changes within OSA leading to dementia. Given the number of overlapping processes, there are a variety of contributory mechanisms leading to or aggravating underlying dementia. TIA: Transient ischemic attack. The figure adapted from [4], with permission from Elsevier, 2016.

Obstructive Sleep Apnea (OSA) occurs in 7% of the adult population and up to 7.5% of the pediatric population. Clinically, OSA is characterized by intermittent apnea/hypopnea, arousals, and sleep fragmentation; however, the clinical symptoms vary greatly between patients (Figure 1). OSA is a common heritable disorder, but a few genetic loci and risk genes are reported from the previous studies. For example, Chen et al. performed a multiethnic meta genome-wide association analysis and found a possible association between the apnea-hypopnea index and rs12936587, which is on chromosome 17 and is overlapped with RAI1 (Retinoic Acid Induced 1) [1]. OSA has been correlated with symptomatology and disease expression in adults with dementia and is associated with several neurological diseases including stroke, as well as neurocognitive and behavioral symptoms. Untreated OSA in the adult population has been shown to accelerate dementia progression while treatment with continuous positive airway pressure (CPAP) has been shown to slow disease progression to varying degrees [2,3].

However, demonstration of causality is difficult, as dementia in the absence of OSA is known to cause abnormalities in sleep structure. Furthermore, sleep fragmentation is known to be an early feature of Alzheimer’s disease and is independently associated with disease progression and intensity [5]. Therefore, as treatment of OSA may impact the progression of dementia, it is important to delineate common gene drivers between these seemingly interrelated disease processes. These would provide clinical biomarkers which could be potential therapeutic targets. To date, characterizing common genomic features between the two, despite several studies independently identifying loci of disease in both groups, has not been elucidated [1,6]. Therefore, we designed a systems biology approach to identify common genes associated with both dementia and OSA. This approach uses network-based identification of common driver (NICD) genes of multiple phenotypes or disease-associated phenotypes utilizing a sequence of techniques involving bioinformatics, public biomedical databases, and computational biology. Specifically, NICD uses the Parsimonious Composite Network (PCNet), which is a composite network created by combining 21 gene–gene interaction networks such as STRING [7], GIANT [8], and ConsensusPathDB [9], and is focused on reducing the false positives of novel disease gene identification [10] and DisGeNET, which is a database which identifies associations between diseases utilizing computational mining techniques classified by evidence [11] to identify the common genes that are associated with multiple diseases.

The aim of this study was to identify driver genes that contribute to disease pathogenesis for dementia and OSA.

## 2. Methods

**Figure 2:**
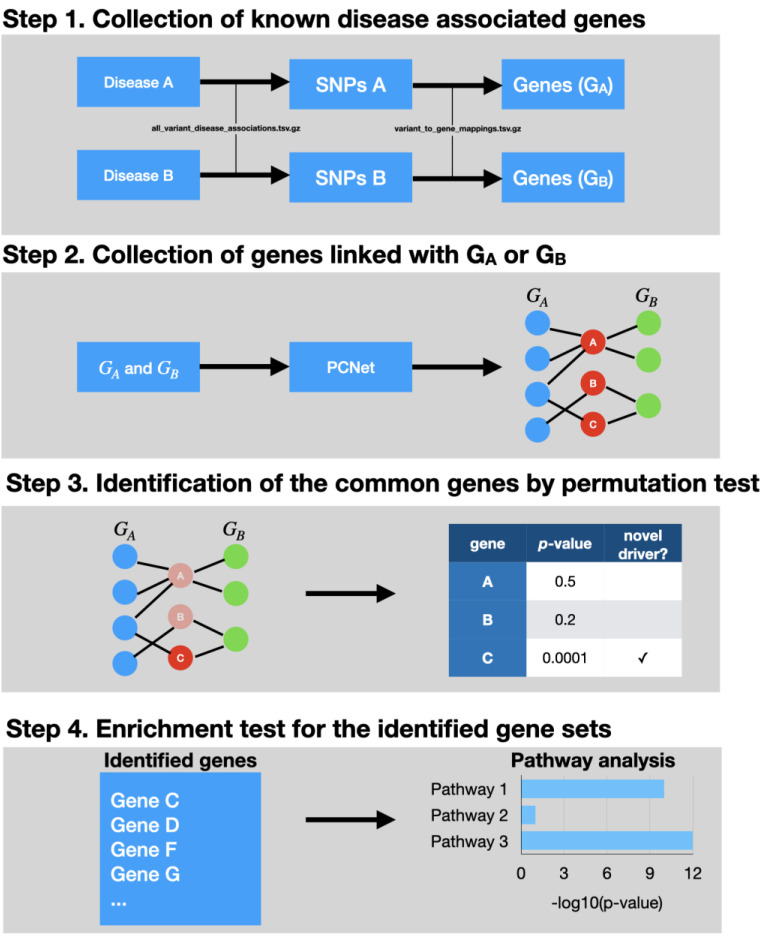
This figure demonstrates the network-based identification of common driver genes (NICD) workflow, with the specific data sets and programs used construct the algorithm. In the topmost panel, the diseases and genes are identified. These are then placed into PCNet, which is a composite gene network focused on common gene identification for query. In order to remove those genes which maybe randomly associated, a permutation assay with Benjamini–Hoichberg correction is done to ensure that proper candidates demonstrate strength of association. In the bottom-most panel, an enrichment assay is done on the candidate genes which tests their association with canonical pathways.

The preparation and validation of NICD was a multistep process (Figure 2).

### 2.1. Biological Network Preparation

NICD is a computational approach and requires a biological network and multiple gene sets for each disease of interest. The biological network is used to detect genes and is physically linked with databases of gene sets. In this study, we used PCNet v1.3 [10]. PCNet is a composite network and was constructed for the purpose of identifying common diseases genes. We downloaded the network from the National Data Exchange (NDEx UUID: f93f402c-86d4-11e7-a10d-0ac135e8bacf). We excluded the Ubiquitin C (UBC) gene from the network as this gene connects with nearly every other gene and therefore causes significant artifacts during analysis. Furthermore, the ubiquitous nature of this gene is not causally tied to a single disease mechanism. We then focused on performing permutation assays between the genes collected from both diseases, utilizing the workflow described in Figure 2.

### 2.2. Collecting Disease Gene Sets of OSA and Dementia from DisGeNET

To collect disease-specific genes for OSA and dementia, we collected single nucleotide polymorphisms (SNPs) and genes linked with the SNPs from DisGeNET v1.0.0. (https://www.disgenet.org/, accessed on 26 May 2020). DisGeNET is a database which displays associations between diseases and genes or variants that were identified by computation literature mining techniques. The search terms in this database allow the user to search a disease by name and phenotypic association to identify a SNP/gene set of interest. This disease ontology has been utilized in several previous studies [11]. Given disease inputs, NICD first seeks variants that are associated with diseases and maps the variants to genes, and we assumed that genes were associated with a disease/phenotype if the genes had any variant-gene mapping records with any disease/phenotype variants of the disease/phenotype in DisGeNET. For the disease-variant mapping, NICD uses a disease-variant mapping table from the database (all_variant_disease_associations.tsv.gz) and a variant-gene mapping table from the database (variant_to_gene_mappings.tsv.gz). To find the variants in the study, we first used the Unified Medical Language System Concept Unique Identifiers (UMLS CUI) of OSA and dementia in DisGeNET. UMLS CUI is the database identifier in DisGeNET and is used to represent a disease/phenotype. We gathered 14 dementia-related UMLS CUI and three OSA related UMLS CUI (Table 1). Given that OSA has a variety of neurological sequelae (Figure 1), we used a broad category of identifiers in the neurological category to capture as many sequelae as possible.

### 2.3. Candidates of Common Driver Genes Identification

After NICD identifies genes sets for each disease, the next step involves the identification of common driver genes which are linked to both diseases. For each disease gene set, NICD seeks a list of genes that have physical links with any of the genes in the gene set on the biological network, which utilizes human data sets and gene arrays which are publicly available. NICD performs the procedure across the disease gene sets and only retains genes that are expressed in both diseases. The co-expressed genes are considered as “primary candidates”.

### 2.4. Permutation Test to Measure Robustness of Each Primary Candidate Gene

**Figure 3:**
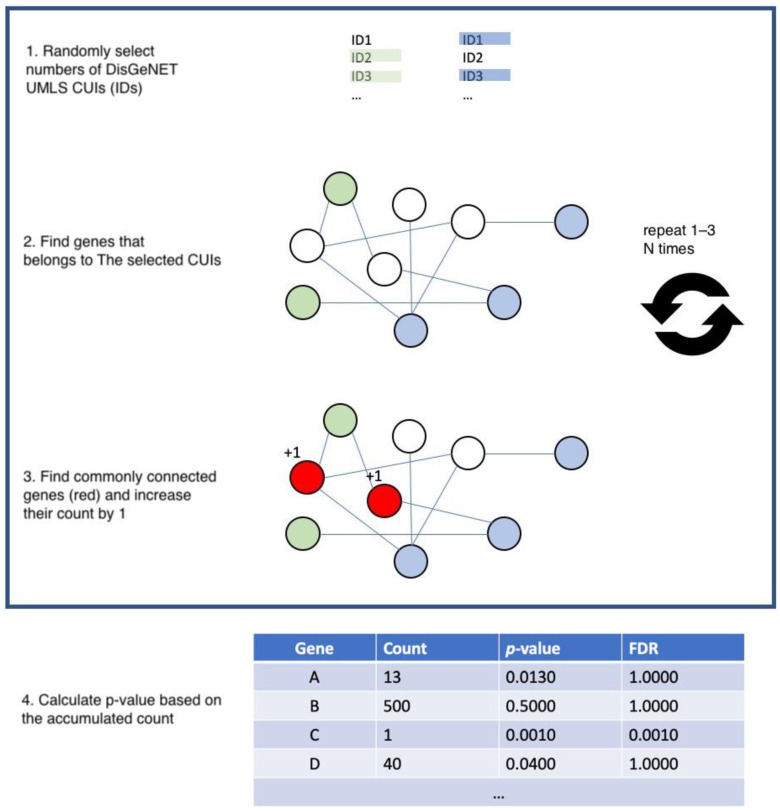
This figure demonstrates the permutation analysis used to identify common driver genes. For a given gene set of interest, this is randomly checked against a list of random genes. This sequence is run repeatedly and corrected to remove overlap and to test the strength of association between genes associated with both diseases. FDR: False Discovery Rate.

To test whether the primary candidates are selected by a random chance, NICD performs a permutation test. A permutation test is a non-parametric statistical approach to empirically estimate the null hypothesis by a random shuffling manner. It has been widely used for many bioinformatics studies such as epistasis [12], multi-omics data integration [13], and gene–gene network construction [14]. The permutation test of NICD is as follows; NICD randomly picks the same number of genes and performs an identification assay. Overlapping between the random gene list and the acquired gene list could be due to random chance. However, while it is possible to identify overlap between the candidates and genes by chance, the findings may not be completely random. The possibility is exponential, so it is not computationally possible to calculate the probability of randomness of primary candidates. Instead of enumerating every possibility, NICD performs a permutation test as described in Figure 3.

1. Let A be the number of DisGeNET IDs where A is the genes identified from disease A;
2. Let B be the number of DisGeNET IDs where B is the genes of the disease from disease B;
3. Let C be the genes that are common genes of both sampled ID sets.
4. For each gene in C, increase the count by 1;
5. Perform Step 1–4 for 100 × *n* times where *n* is the array of common genes from the previous step (which was not chance);
6. For each of the common genes, calculate *p*-value by x/100 × *n*, where x is the gene count for step 1–5;
7. Correct the *p*-value using Benjamini–Hochberg correction to decrease the number of false positives.

After performing the permutation test, a list of common genes is produced. The code utilized for performing this test is available in the link for data sharing at the end of the manuscript.

### 2.5. Gene Enrichment Analysis

To understand the biological mechanisms of input genes or the potential common drivers, NICD performs gene enrichment analyses. During the analyses, NICD uses Enrichr (https://amp.pharm.mssm.edu/Enrichr/, accessed on 26 May 2020) to test enrichment between genes and any canonical genes sets that are defined by gene ontologies or pathway annotations [15,16]. This allows us to understand the mechanistic underpinning of a disease process.

## 3. Results

**Figure 4:**
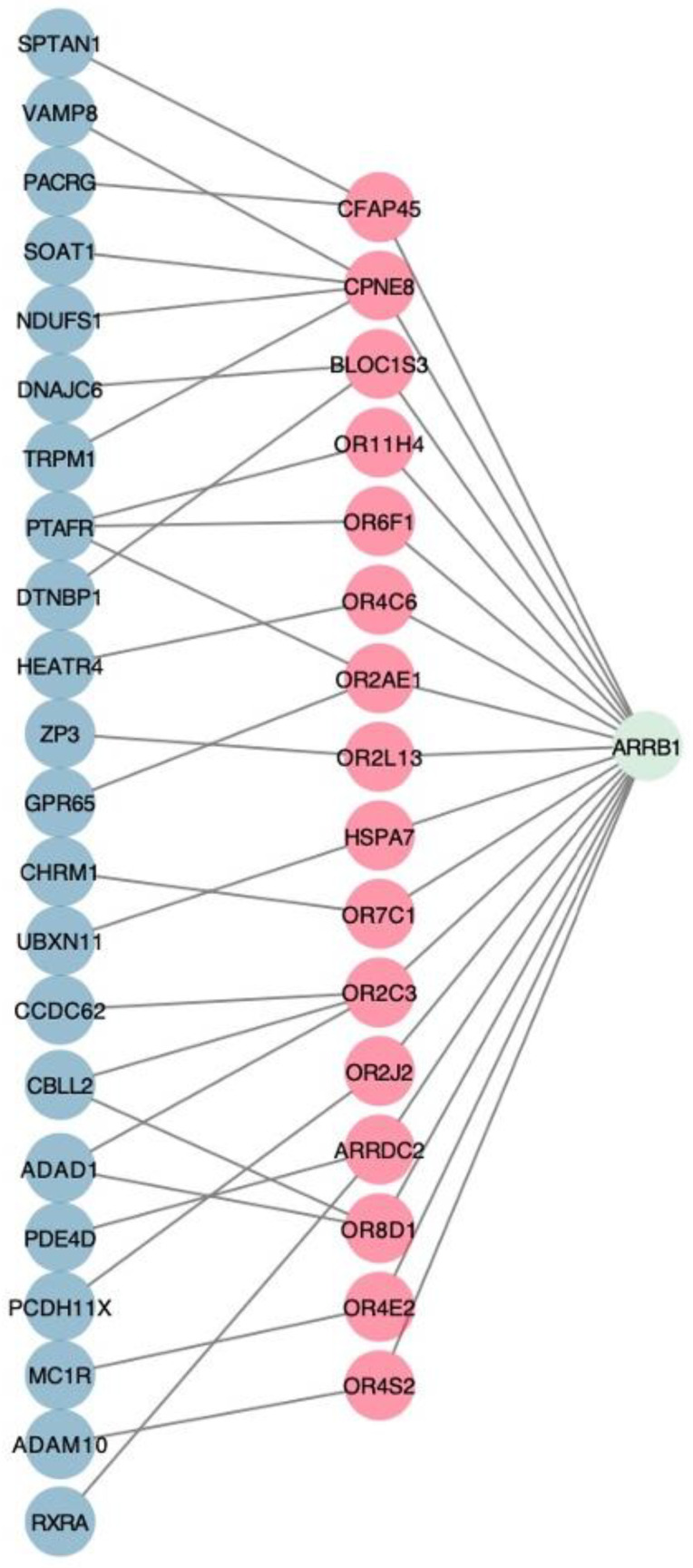
The network of common driver genes that are associated with olfactory transduction and GPCR. Red nodes correspond to the common drivers and blue nodes and green nodes correspond to dementia genes and OSA genes, respectively. This represents the fundamental premise behind identifying common gene drivers common to both disease processes.

**Figure 5:**
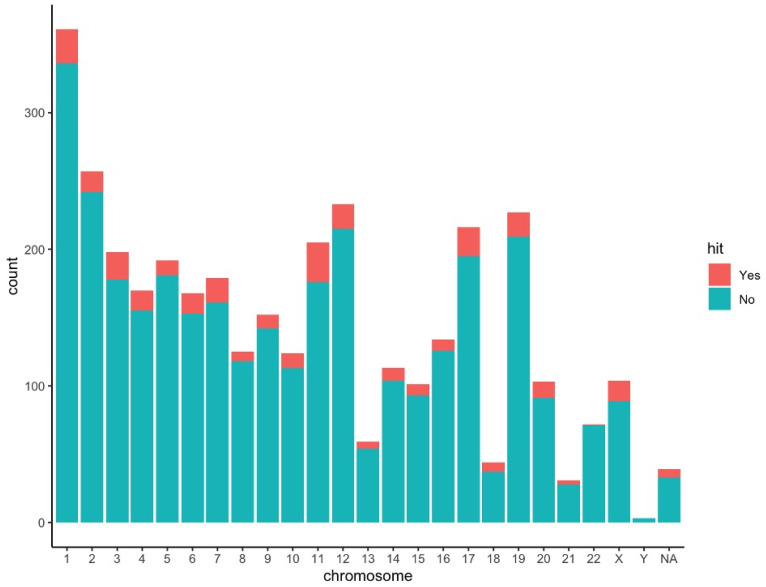
Distribution of commonly linked genes (green and red) and identified common drivers (red) in each human chromosome.

Initially, 1108 dementia-associated and 23 OSA-associated genes were identified. The aforementioned genes were used to identify 3610 genes which had links with both any OSA-associated genes and any dementia-associated genes. Following NICD with performance of permutation testing, 42 genes were identified with common associations between Alzheimer’s disease and OSA (Table 2). These identified genes by NICD were then mapped back to the pathway which the gene affects. Many of the genes were involved with G-coupled protein receptor (GPCR) and olfactory signaling pathways. Gene enrichment analysis using Enrichr found that a subset of genes (10/42) was significantly enriched in these pathways (*p* = 1.314 × 10^−9^ for olfactory transduction in the KEGG 2019 pathway library and *p* = 0.00002605 for signaling by GPCR in the BioPlanet 2019 pathway library) (Figure 4). The identified gene candidates were then characterized by chromosome (Figure 5).

With regards to single nucleotide polymorphisms, we used the data from two large genome wide association study (GWAS) repositories which have been previously published [1,5]. No common SNPs between the two diseases were identified or achieved statistical significance.

## 4. Discussion

OSA has long been associated with cognitive impairment and depression [13], and neurological disorders have been associated with sleep disruptions. There are proposed shared pathophysiological mechanisms between OSA and dementia [17] which have been demonstrated in a large clinical study as well [18]. Specifically, Alzheimer’s diease patients treated for OSA demonstrated increased slow wave sleep (SWS) as well as greater clearance of Aβ protein, the quantity of which has been directly correlated with disease severity. However, patients with dementia often have compliance difficulty with the use of CPAP resulting in [19] a bidirectional, positive feedback loop leading to worse outcomes [20].

The identified gene drivers in our study focused on two pathways: (1) olfaction and (2) GPCR. Olfactory pathways in dementia have drawn significant interest due to their early involvement in the dementia process [21]. Loss of olfaction is a common feature in several types of dementia with proteomic signatures varying across dementia phenotypes [22]. It has been suggested that the olfactory bulb could be the nidus of pathology in several dementia subtypes [23]. In OSA, disorders of olfaction have been characterized as well, with the degree of olfactory disturbance correlating with the severity of disease [24]. Furthermore, there is improvement in olfaction after therapy with OSA [25], demonstrating that olfactory pathways maybe early sites of involvement with both diseases.

The role of GPCRs in the context of both diseases is less clear due to the subtypes and nearly ubiquitous nature of these receptors. Multiple pathological variants have been described for GPCRs for several different clinical phenotypes of dementia [26,27,28]. There have been no studies to date studying GPCRs in the setting of underlying OSA. Unlike dementia, OSA is a systemic disease process with multiple sites of end organ involvement, thereby making localized study difficult.

While this study can identify drivers of both diseases, we are unable to determine what the triggers for those drivers are. Additionally, we are unable to determine causality, which would require more in depth translational and clinical studies to delineate. However, this study can demonstrate that there are gene drivers commonly linked between OSA and dementia. This methodology can be further applied to other disease pairs which are independent but may lead to worsening of the other disease’s clinical presentation. Examples include Type 2 diabetes mellitus and hypertension, as well as more hereditable diseases, such as obesity and alcoholism.

In summation, this study provided a new framework for identifying common gene drivers, utilizing several known techniques combined with permutation analysis. This algorithm was used to identify common drivers between two often co-existing but mutually exclusive diseases, OSA and dementia. These common gene drivers can then be tested for clinical utility and potentially be used as biomarkers after validation. This particular methodology is particularly useful to identify drivers for diseases which have different etiologies but can co-exist and can create care issues when comorbid. This can facilitate driving therapies which can impact multiple disease processes in the same patient.

### Key Points

Question: Is there a method to identify common gene drivers between two diseases that are comorbid but unrelated, specifically OSA and dementia?

Findings: This study describes a common methodology that utilizes publicly available data to identify 42 common genes between OSA and dementia

Meaning: This methodology can be extrapolated to multiple large data sets to potentially identify gene drivers of common, comorbid, but independent diseases.

## Figures and Tables

**Table 1:** The list of DisGeNET disease/phenotype identifiers (Unified Medical Language System Concept Unique Identifiers, UMLS CUI) used in the study.

Category	UMLS CUI	Disease/Phenotype Name
Dementia	C0524851	Neurodegenerative Disorders
	C0002736	Amyotrophic Lateral Sclerosis
	C0030567	Parkinson Disease
	C0002395	Alzheimer’s Disease
	C0020179	Huntington Disease
	C0497327	Dementia
	C0338656	Impaired cognition
	C0011265	Presenile dementia
	C0038454	Cerebrovascular accident
	C0242422	Parkinsonian Disorders
	C0233794	Memory impairment
	C0026769	Multiple Sclerosis
	C0338451	Frontotemporal dementia
	C0752347	Lewy Body Disease
OSA	C0520679	Sleep Apnea, Obstructive
	C0037315	Sleep Apnea Syndromes
	C0520680	Sleep Apnea, Central

**Table 2:** The list of common driver genes identified by NICD.

Common Gene	Dementia Genes	Osa Genes	Olfactory	GPCR	*p* Value
TMEM181	SH3RF1	NRG1			1.36 × 10^−4^
TSPAN19	TSPAN16	TSPAN18			8.31 × 10^−6^
CSTL1	AHSG, PRKN	NRG1			4.93 × 10^−4^
RNF121	ITGB2	LPAR1			2.60 × 10^−4^
AL121594.3	RNF4	NRG1			5.04 × 10^−4^
OR4E2	MC1R	ARRB1	✓	✓	5.15 × 10^−4^
FAM241A	PLP1, LINGO1	LPAR1, SGCD			2.35 × 10^−4^
FITM2	CCRL2, HTR2C	LPAR1, TSPAN18			2.74 × 10^−4^
OR2L13	ZP3	ARRB1	✓	✓	2.02 × 10^−4^
OR11H4	PTAFR	ARRB1	✓		2.13 × 10^−4^
CST11	CST3, AHSG	NRG1			3.43 × 10^−4^
OR4C6	HEATR4	ARRB1	✓	✓	2.71 × 10^−4^
TMEM120A	VKORC1	TSPAN18			2.47 × 10^−4^
OR2AE1	PTAFR, GPR65	ARRB1	✓	✓	4.74 × 10^−4^
OR7C1	CHRM1	ARRB1	✓	✓	3.63 × 10^−4^
TMEM87B	SLC30A7	LPAR1			1.91 × 10^−4^
OR8D1	CBLL2, ADAD1	ARRB1	✓	✓	2.88 × 10^−4^
OR4S2	ADAM10	ARRB1	✓	✓	3.57 × 10^−4^
OR2J2	PCDH11X	ARRB1	✓	✓	5.26 × 10^−4^
TMED8	TMED9	PTGER3			1.22 × 10^−4^
OR2C3	CBLL2, ADAD1, CCDC62	ARRB1			4.16 × 10^−4^
TMCO5A	DKKL1, TEX33, ADAD1, CCDC62, CBLL2	TSPAN18			3.38 × 10^−4^
HSPA7	UBXN11	ARRB1			2.27 × 10^−4^
ZSWIM9	ESR2, ZNF292	AHDC1			5.15 × 10^−4^
FABP9	FABP2	FABP4			6.09 × 10^−5^
GTSF1	HSPB1	MPHOSPH6			1.47 × 10^−4^
TMEM151A	CAMK2A, SNCB	LPAR1			5.60 × 10^−4^
TMEM218	NARS2	TSPAN18			3.99 × 10^−4^
MGAT5B	FAM171A2, MGAT5	PTGER3			2.44 × 10^−4^
OR6F1	PTAFR	ARRB1	✓	✓	2.13 × 10^−4^
ZNF385D	ZNF804A	PTGER3			4.71 × 10^−5^
TMEM161B	RPS6KB1	LPAR1			1.63 × 10^−4^
LARGE2	DKKL1	NRG1			4.13 × 10^−4^
SCFD2	SCFD1, STXBP2, UBE2Z, SIRT2	NBAS			2.83 × 10^−4^
RAVER2	CNKSR3	NRG1			2.94 × 10^−4^
CPNE8	TRPM1, VAMP8, NDUFS1, SOAT1	ARRB1			5.29 × 10^−4^
ARRDC2	RXRA, PDE4D	ARRB1			4.68 × 10^−4^
OLFM2	TUBB1	NBAS			3.55 × 10^−4^
ENOX1	NOX4, SETD1A, RBMS3	LPAR1			5.76 × 10^−4^
TMEM120B	RNF5, SIGMAR1	LPAR1			4.32 × 10^−4^
BLOC1S3	DTNBP1, DNAJC6	ARRB1			2.63 × 10^−4^
CFAP45	SPTAN1, PACRG	ARRB1			4.13 × 10^−4^

## Data Availability

DisGeNET database is available at https://www.disgenet.org/downloads (accessed on 26 May 2020). PCNet is available at http://www.ndexbio.org/ (accessed on 26 May 2020). The processed data used in the study are available at https://github.com/hyunhwan-jeong/NICD (accessed on 8 April 2021).

## Software Availability

NICD software is written in python3 and available at https://github.com/hyunhwan-jeong/NICD (accessed on 8 April 2021).
